# Effect of Physiotherapy on a Rare Case of Malunion of Femur and Patellar Fracture in a 43-Year-Old Male: A Case Report

**DOI:** 10.7759/cureus.49239

**Published:** 2023-11-22

**Authors:** Ishwin Kaur B Bagga, Deepali S Patil, Medhavi V Jagzape

**Affiliations:** 1 Musculoskeletal Physiotherapy, Ravi Nair Physiotherapy College, Datta Meghe Institute of Higher Education & Research, Wardha, IND

**Keywords:** case report, quality of life, rehabilitation, physical therapy, gait training, myofascial release, strengthening, comminuted patellar fracture, supracondylar femur fracture

## Abstract

The hip is a ball-and-socket joint surrounded by strong and well-balanced muscles that allow for a wide range of motion in many physical planes. Iliofemoral, ischiofemoral, and pubofemoral are the three major ligaments of the hip joint that provide stability to the joint. Supracondylar femoral fractures are common in old age and can be caused in young people due to accidents or traumatic causes. These types of fractures are complicated to fix surgically due to different architectural designs. If not treated appropriately, these can cause malunion or non-union of the joint. The knee joint is a synovial joint of the hinge type. It has two major degrees of movement, which are flexion and extension. However, rotation in both the medial and lateral directions is possible to some extent in the joint. Patellar fractures can be transverse, vertical, comminuted, marginal, or osteochondral. In this case report, we present a 43-year-old male patient who had a history of falling from a bike. He was diagnosed with a comminuted supracondylar fracture of the left femur and a comminuted fracture of the patella on the left side on an X-ray. For this, he was managed with open reduction, internal fixation, and vacuum-assisted closure (VAC). Physiotherapy rehabilitation was programmed to attain a good and fast recovery for the patient to make him functionally independent and improve his quality of life.

## Introduction

Hip joint

The hip is an actual ball-and-socket joint encircled by strong muscles [1]. The hip joint has four diarthrodial or synovial joint characteristics: it contains joint surfaces covered with articular cartilage, a joint cavity, is enclosed by a ligamentous capsule, and has a synovial membrane generating synovial fluid [1]. The hip joint is supported by three major fibrous capsular ligaments (iliofemoral, ischiofemoral, and pubofemoral), each of which performs a unique functional role in joint stabilization [2-4].

Fractures of femur

Supracondylar femur fractures are serious injuries that might be difficult to repair surgically [5]. Distal femur fractures account for about 3%-6% of all femur fractures and 1% of all fractures [6,7]. Because of the distal femur's unusual architecture and closeness to the knee joint, surgical healing of these fractures is difficult. Because of the muscular traction of the gastrocnemius and adductor muscles, fractures frequently exhibit characteristic displacement patterns [6]. Regardless of the cause, distal femoral fractures are frequently complex, intraarticular, and comminuted, making attaining and maintaining an appropriate reduction difficult. To lessen the possibility of nonunion, special care must be taken to prevent disturbing the soft tissue envelope [8,9].

Knee joint and patellar fractures

The knee is a complicated, modified hinge joint. It moves most in flexion and extension in the sagittal plane [10]. Patella increases the quadriceps moment arm [11,12]. The patella's articular cartilage is the thickest in the body. The patella's articular surface has facets that vary in size and direction in individuals [11,13]. Patellofemoral cartilage is a biphasic material that consists of a freely flowing fluid phase and a porous phase [11,14]. Traumatic patella fractures can be transverse, vertical, comminuted, marginal, or osteochondral [15]. Comminuted fractures are common in individuals who have been damaged in several ways. These instances frequently manifest with extensive soft tissue injuries. Non-displaced fractures are those with a displacement of less than 3 mm [15,16].

## Case presentation

Patient information

A 43-year-old male with right-hand dominance and driver by occupation had a fall from his bike on 24th August 2023. The local people on the site of the accident called an ambulance and took the patient to Wardha, where first aid was done, and then he was brought to Acharya Vinobha Bhave Rural Hospital casualty on the same day at 3 pm where various investigations were done, such as blood investigations, X-ray and MRI. An X-ray revealed a comminuted supracondylar fracture of the left femur and a comminuted fracture of the patella on the left side, which was managed with open reduction and internal fixation, and vacuum-assisted closure (VAC) was applied on 26th August 2023. After four days, the patient was taken to OT (operating theatre) for the removal of VAC and wound debridement, and the wound was sutured. The patient was referred for physiotherapy and rehabilitation. Girth measurement was done for both thighs, and it was found that the girth over the right knee is 85cm, and the girth over the left knee is 93cm. This signifies that there is muscle wasting in the left lower limb. Table 1 shows the findings of range of motion (ROM) for both the lower limb, pre-intervention, and post-intervention; the symbol ° represents the degree of range of motion. Table 2 shows the grade of manual muscle testing for both the lower limb, pre-intervention, and post-intervention.

**Table 1:** Range of Motion ° represents the range of motion

Joint	Right	Left		
		Pre-intervention	Post-intervention Active & Passive	
Hip:				
Flexion	0-120°	0-90°	0-110°	0-120°
Extension	0-20°	0-10°	0-20°	0-20°
Abduction	0-20°	0-20°	0-20°	0-20°
Knee:				
Flexion	0-125°	0-30°	0-80°	0-85°
Extension	125°-0	30°-0	80°-0	85°-0
Ankle:				
Plantarflexion	0-30°	0-20°	0-25°	0-30°
Dorsiflexion	0-20°	0-10°	0-15°	0-20°

**Table 2:** Manual Muscle Testing

Joint	MMT grade (right)	MMT grade (left)	
		Pre-intervention	Post-intervention
Hip:			
Flexors	5	3	4
Extensors:	5	3	4
Abductors:	5	3	4
Knee:			
Flexors	5	3	4
Extensors	5	3	4
Ankle:			
Plantarflexors	5	4	4
Dorsiflexors	5	4	4

Investigations

**Figure 1:**
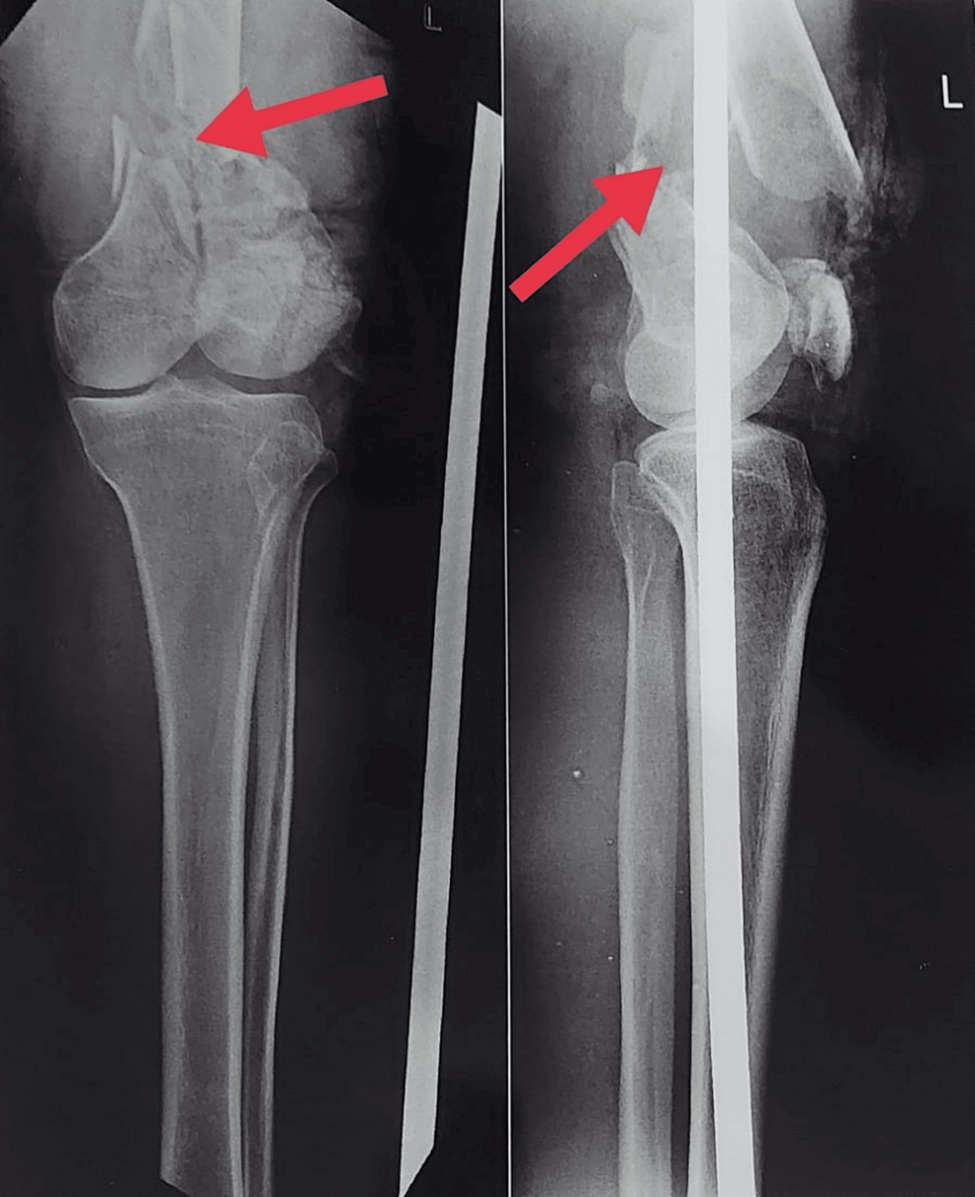
Pre-operative X-ray in posterior and lateral view (According to the AO classification the fracture is classified in type C2 which is supracondylar comminuted fracture of femur) Red arrow shows the fracture site in posterior and lateral view

**Figure 2:**
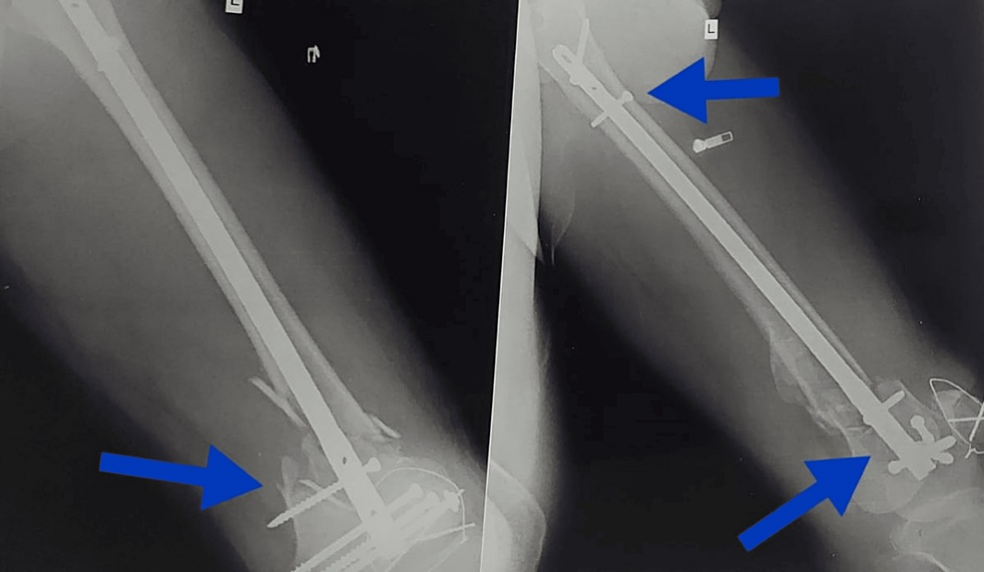
Post-operative X-ray (Femur interlock nail with cannulated screw fixation and patella encircling wire) Blue arrows shows the location of cannulated screw fixation and patella encircling wire

**Figure 3:**
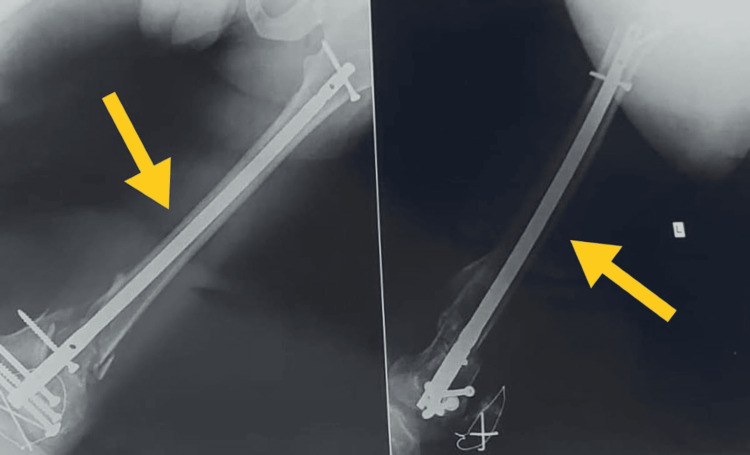
Current X-ray showing malunion and non-union of the bone Yellow arrow shows the malunion of bone

Figure 1 shows the pre-operative X-ray in posterior and lateral view. Figure 2 shows the post-operative X-ray, and Figure 3 shows current X-ray showing malunion and non-union of bone. 

Outcome measures

Table 3 shows the outcome measures taken for the study and the scores pre- and post-intervention. The symbol ° represents the degree of range of motion. 

**Table 3:** Outcome measures with score

Variables	Pre-intervention	Post-intervention
Lower Extremity Functional Scale	11.25%	46.25%
ROM for Knee Flexion	0-30°	0-80°
Manual Muscle Testing for knee flexors	Grade 3	Grade 4
	Score	Prediction
Barthel Index	65/100	Indicates patient is moderately dependent
Numerical Pain Rating Scale	On rest: 3/10 and On activity: 5/10	______

Physiotherapy management

**Figure 4:**
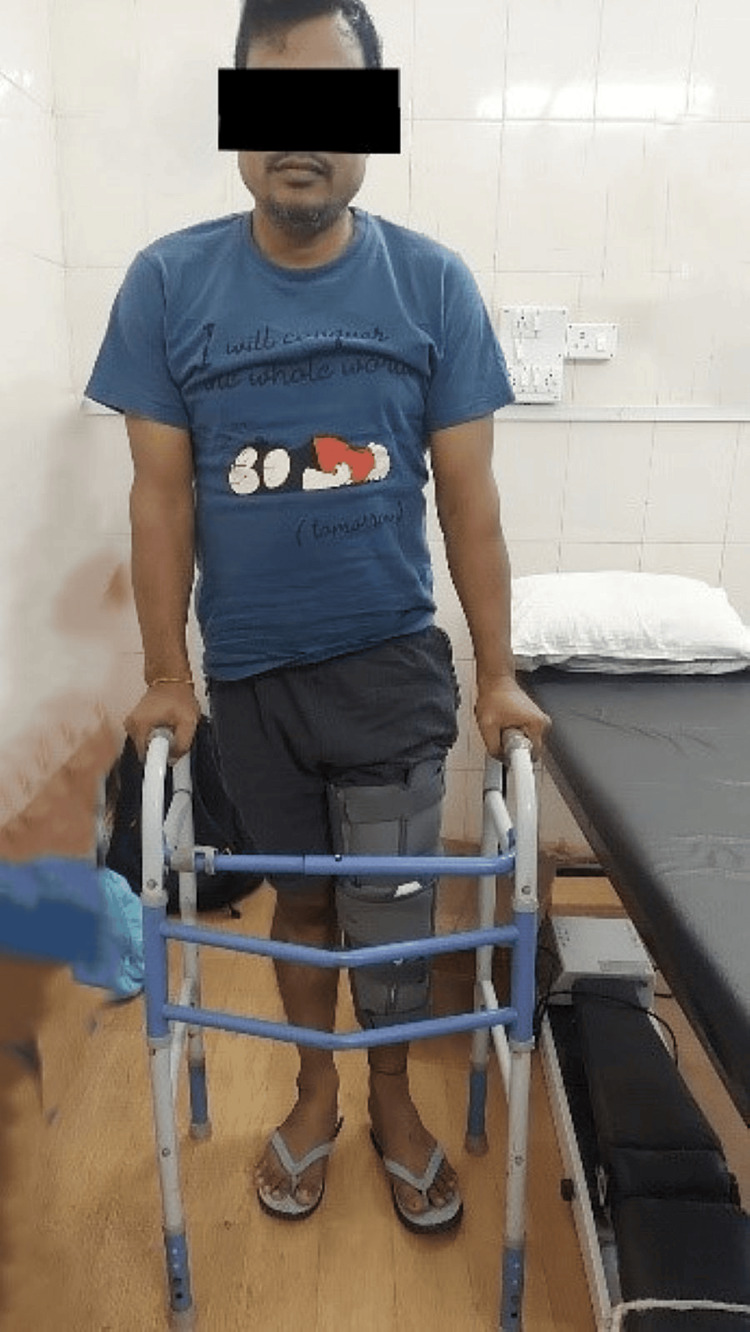
Ambulating patient with brace and walker

Goals: Patient education: The therapist will educate the patient about his condition and will explain to him how taking physiotherapy can help him in a faster recovery. He will explain to the patient how physiotherapy can make him functionally independent. To reduce pain: To reduce pain use of cryotherapy will be done. Later, the application of therapeutic modalities can also help in reducing pain. To improve the range of motion of the knee: The use of continuous passive motion (CPM) can be done to help improve the ROM of the knee flexion and extension. To improve the strength of knee musculature: Strengthening exercises to the knee musculature to improve the strength around the knee joint are given to the patient. Strengthening to the hamstrings and straight leg raise exercises along with heel slides are given. To improve functional mobility: Gait training with the use of a walker and knee brace is done. Postural correction exercises are taught to the patient. This helps to improve the overall functional mobility of the patient. Table 4 shows the treatment protocol from 1st day to the 15th day. Table 5 shows the treatment protocol from the 15th day to the 30th day. Figure 4 shows ambulating the patient with the help of a knee brace and walker.

**Table 4:** 1st to 15th Day

Treatment	Dosage	Rationale
Static quadriceps	15 repetitions with 5-10 second hold	To initiate the action of quadricep muscles
Static hamstrings	15 repetitions with 5-10 second hold	To initiate the action of hamstring muscles
Static abdominals	15 repetitions with 5-10 second hold	To initiate the action of abdominals
Continuous passive motion (CPM)	For 30 minutes, initially start with 30 degrees	To maintain and increase the knee range of motion
Straight leg raise (With brace)	10 repetitions with 5-10 second hold	To strengthen the hip muscles
Prone knee hang	10 repetitions	To reduce the extension, lag of the patient
Heel slides	10 repetitions	To maintain the range of knee flexion

**Table 5:** 15th to 30th Day

Treatment	Dosage	Rationale
Knee Mobilization	5 repetitions with 5-10 second hold	To maintain and increase the range of motion
Vastus Medialis Oblique Strengthening	With 5 second hold	To improve the range of terminal extension
Sit to Stand with walker	10 minutes	To improve and maintain balance
Myofascial Release	10 minutes	To release the tight fascia
Gait Training	10 minutes	Ambulate the patient

## Discussion

Internal fixation of displaced supracondylar periprosthetic femur fractures in the presence of a stable knee prosthesis is the suggested treatment approach to ensure stability and alignment to allow the patient to mobilize sooner [17,18]. Because of the short distal fragment and osteoporotic bone in older patients with many comorbidities, surgical treatment of these fractures is difficult [17-19]. The two most prevalent surgical treatment methods are locked plating and retrograde intramedullary nailing (RIMN) [17,18]. In cases of comminuted patellar fractures, because of the tiny size of the fragment and the comminuted fracture pattern, finding an optimum therapy that consistently yields excellent outcomes is difficult [20]. The tension band approach, the most generally used method for transverse patellar fracture repair, may not produce appropriate stability in handling inferior patellar pole fractures [20]. Plate attachment may be regarded as an effective method for overcoming the drawbacks of tension band wiring [20-22]. The patient in this report was given a physical rehabilitation intervention plan, which was planned by a qualified musculoskeletal physiotherapist. Protocol with static quadriceps, hamstrings, and abdominals, along with straight leg raises with braces, was initiated on the first day of the rehabilitation program. The use of continuous passive motion (CPM) and vastus medialis oblique (VMO) strengthening was done to increase the ROM and to prevent stiffness and tightness. Heel slides and ankle pumps were given to reduce swelling in the ankle and help improve knee range of motion. For malunion of bones treatment, knee mobilization and strengthening exercises were given to the patient. The goal of physical therapy was to prevent secondary complications. The goal of the above case study was to highlight the importance of physiotherapy rehabilitation in achieving long-term effects in patients and improving their quality of life by making them functionally independent.

## Conclusions

In conclusion, physiotherapy intervention after surgical intervention is found to be beneficial in improving the ROM and strength of the patient. An accurate physiotherapy plan was designed for the patient in which he was given knee mobilization, strengthening exercises, and heel slides, which helped in his early ambulation. Physiotherapy also plays a crucial role in the treatment of bone malunion. Physiotherapists help individuals regain strength, flexibility, and function in their injured limbs through targeted exercises, mobilization techniques, strengthening exercises, manual therapy, and patient education, ultimately improving their chances of a successful and fulfilling rehabilitation journey. Physical therapy helps to improve the overall quality of life of the patient and helps him restore his activities of daily living.
